# Focusing on the AhR: A Potential Mechanism for Immune Effects of Prenatal Exposures

**DOI:** 10.1289/ehp.122-A313

**Published:** 2014-11-01

**Authors:** Lindsey Konkel

**Affiliations:** Lindsey Konkel is a Worcester, MA–based journalist who reports on science, health, and the environment. She is an editor for *Environmental Health News* and *The Daily Climate*.

Epidemiological studies suggest that prenatal and early-life exposures to certain chemicals can influence immune system function later in life.^1^ However, the mechanisms by which these changes may occur remain unknown. In this issue of *EHP*, researchers report an altered immune response to influenza virus infection in adult mice that had been exposed prenatally to 2,3,7,8-tetrachlordibenzo-*p*-dioxin (TCDD), revealing a novel cellular target of developmental exposures.^2^

**Figure:**
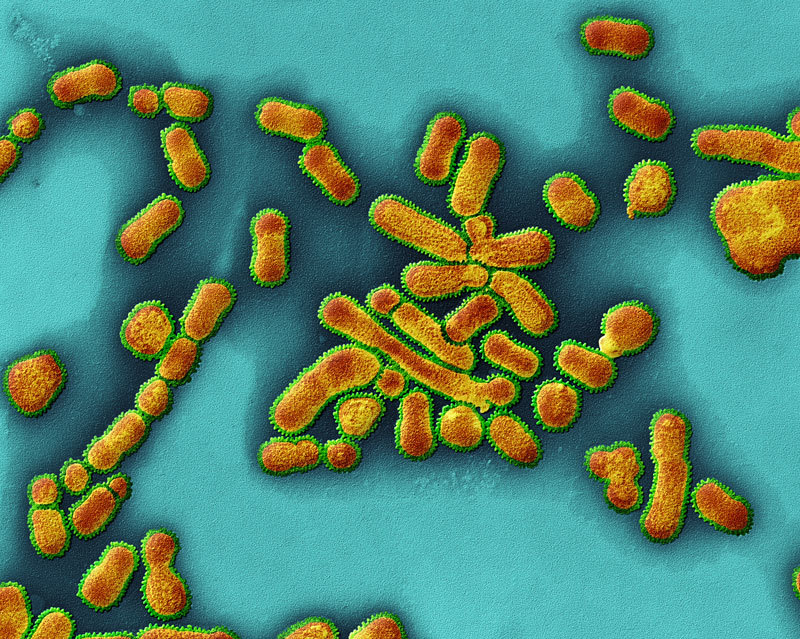
A new study of influenza A (shown) and TCDD provides a mechanistic framework that may help researchers understand how certain chemical exposures affect the developing immune system. © Eye of Science/Science Source

“Researchers have been demonstrating for a number of years that the developing immune system is uniquely sensitive to chemical exposures,” says Dori Germolec, leader of the Systems Toxicology Group at the National Institute of Environmental Health Sciences. “This study provides a mechanistic framework that may help us understand why this is the case.” Germolec was not involved in the current study.

Previous epidemiological studies have reported that maternal and cord blood levels of polychlorinated biphenyls and dioxins corresponded with decreased immune response to routine vaccinations^3^ and increased respiratory infections in children.^4^ The new study is thought to be the first to demonstrate that CD4^+^ T cells—immune cells that are critical in the creation of effective responses to both vaccinations and infections—are functionally altered after developmental exposure to a member of the same family of chemicals as those measured in the epidemiological literature.

TCDD is a bioaccumulative by-product of industrial processes such as waste incineration and pesticide production.^5^ The compound has been shown in human and animal studies to alter transcription of genes by binding to the aryl hydrocarbon receptor (AhR).^1^

The researchers exposed pregnant female mice to an environmentally relevant dose of TCDD (1 μg/kg body weight). At age 6–8 weeks, the adult offspring were infected with influenza A virus.

CD4^+^ T cells have the ability to differentiate into distinct subsets of immune cells in response to infection. Compared with mice born to untreated mothers, mice exposed to TCDD *in utero* showed a reduced frequency of four subsets of conventional helper CD4^+^ T cells, which are responsible for enhancing the antiviral immune response. Additionally, says senior study author B. Paige Lawrence, an immunotoxicologist at the University of Rochester, “we saw an increase in the proportion of regulatory T cells, which put the brakes on an immune response.”

To test whether developmental exposure to TCDD caused intrinsic changes in the ability of CD4^+^ T cells to differentiate, the researchers transferred purified cells from exposed offspring into unexposed mice. They observed a reduction in the number of T helper cells responding to influenza virus infection when the cells came from exposed mice. Additionally, exposed mice had two- to three-fold fewer B cells, which are responsible for the antibody response, than control mice.^2^ The changes persisted, suggesting that “developmental exposure to chemicals may change epigenetic programming of the immune cells, such that their response to infection is durably altered,” Lawrence says.

Future studies will explore whether changes in immune function can be transferred to future generations of offspring. “The idea that you can expose a pregnant mouse to an environmental chemical like a dioxin during pregnancy and have that adversely impact the immune response [of its offspring] to influenza later in life has profound consequences for public health,” says Michael Laiosa, an environmental health scientist from the Zilber School of Public Health, University of Wisconsin–Milwaukee, who was not involved in the study. Laiosa notes it’s not clear whether all chemicals that bind the AhR would have the same effect as TCDD, which is one of the most potent agents known to act on that receptor.

New evidence in mice suggests that brominated dioxin-like compounds that bind to the AhR also affect immune function.^6^ If similar immunomodulatory effects occur in humans, we may not be recognizing the impact these bioaccumulating compounds have on the overall burden of disease, particularly in groups with higher exposures, Germolec says.

Previous studies investigating the impact of chemical exposures on the human immune system have focused largely on antibody levels.^3^^,^^7^^,^^8^ This new work “has implications for examining how early-life exposures affect immune function in the human population, where antibody responses are often the sole measurement,” the authors wrote.^2^ Indeed, Lawrence now hopes to turn this new knowledge toward studying human populations. She says, “This tells us we should also be looking more closely at the function of different cell types and what those cells contribute to the immune response—not just measuring the level of antibodies produced.”
